# Assessment of Drug Dosing Appropriateness in Hospitalized Chronic Kidney Disease Patients with Cardiovascular Diseases: A Cross-Sectional Study in the Al-Baha Region, Saudi Arabia (2023–2025)

**DOI:** 10.3390/jcm15062293

**Published:** 2026-03-17

**Authors:** Lina O. Abdelmagid, Saleh Alghamdi, Mohammad Algarni, Mohammad A. Albanghali, Zuheir Osman, Ahmed Alghamdi, Mohammed Alamri, Mohammed S. Alghamdi, Saeed A. Alzahrani, Fayez Alghamdi, Bassant Mohamed Barakat

**Affiliations:** 1 Faculty of Pharmacy, University of Khartoum, Khartoum 11115, Sudan; olina662@gmail.com; 2 Department of Clinical Pharmacy, Faculty of Pharmacy, Al-Baha University, Al-Baha 65779, Saudi Arabia; saleh.alghamdi@bu.edu.sa (S.A.); maalqarni@bu.edu.sa (M.A.); 3 Department of Public Health, Faculty of Applied Medical Sciences, Al-Baha University, Al-Baha 65779, Saudi Arabia; mohammad.aref@bu.edu.sa; 4 Department of Pharmaceutics, Faculty of Pharmacy, University of Khartoum, Khartoum 11115, Sudan; zuheirosman@uofk.edu; 5 Department of Clinical Pharmacy, College of Pharmacy, King Saud University, Riyadh 11451, Saudi Arabia; aanahi@ksu.edu.sa; 6 Department of Pharmaceutical Care, King Fahad Hospital, Al-Baha 65732, Saudi Arabia; moahalamri@moh.gov.sa; 7 Department of Pharmaceutical Care, Prince Meshari Bin Saud Hospital, Baljurashi, Al-Baha 65639, Saudi Arabia; malghamdi232@moh.gov.sa; 8 Pharmaceutical Care Administration, Al-Baha Health Cluster, Al-Baha 65784, Saudi Arabia; salzahrani56@moh.gov.sa; 9 Department of Academic Affairs, King Fahad Hospital, Al-Baha 65732, Saudi Arabia; fayezsg@moh.gov.sa

**Keywords:** chronic kidney disease, cardiovascular diseases, dose-adjustment, inappropriateness

## Abstract

**Background/Objectives:** For patients diagnosed with chronic kidney disease (CKD), it is important to follow guidelines addressing dose-adjustments for renally eliminated drugs to avoid complications related to toxicity and subtherapeutic effects. In Saudi Arabia, limited data are available regarding appropriate medication doses for CKD. In this study, we investigated the prevalence of inappropriately administered drugs in patients with CKD and examined factors associated with unadjusted renal dosing. **Methods:** A retrospective, cross-sectional, observational analysis (2023–2025) was conducted via a systematic electronic medical record review of hospitalized patients diagnosed with CKD and cardiovascular diseases (CVDs) in the Al-Baha region, Saudi Arabia. Medications were selected and evaluated for appropriate dosing based on creatinine clearance (CrCl). Medications were categorized as appropriately adjusted, inappropriately adjusted, unadjusted, or contraindicated. **Results:** A total of 440 patients (787 prescriptions) were included. At the patient level, 85% had at least one appropriately adjusted medication, 13% had at least one inappropriately adjusted medication, 30% had at least one medication that was not adjusted despite indication, 34% had at least one medication requiring no adjustment, and 17% had at least one contraindicated medication (categories are not mutually exclusive). At the prescription level, which was the primary analytic unit (N = 787), 48% of prescriptions were appropriately adjusted, 7% were inappropriately adjusted, 17% were not adjusted despite indication, 19% required no adjustment, and 10% were contraindicated. Antibiotics accounted for the largest share of inappropriate adjustments, representing 77% (43/56) of all inappropriate dose-adjustment events. In exploratory bivariate analyses, age was not statistically significantly associated with dosing outcomes (Holm-adjusted *p* = 0.145). Polypharmacy was highly prevalent (91% of patients) but was not significantly associated with any dosing outcome in these exploratory analyses, likely due to limited statistical power. **Conclusions:** Our results showed that several regularly prescribed drugs, including metformin, sitagliptin, ceftazidime, ciprofloxacin, and spironolactone, were inappropriately prescribed to patients with CKD. These dosing errors can be avoided by increasing clinicians’ and pharmacists’ awareness of appropriate dosage modifications essential for patients with CKD.

## 1. Introduction

Chronic kidney disease (CKD) is a significant global health problem affecting millions of patients [1,2]. This condition is characterized by abnormalities in kidney structure or function that persist for more than three months and is linked to considerable morbidity and mortality, which leads to a substantial burden on healthcare systems, particularly on hospitalized patients [3,4]. Patients with renal impairment frequently depend on numerous medications to control comorbid conditions and disease-related complications [5,6,7,8,9]. Because many medications are primarily metabolized and eliminated via the renal system, impaired renal function often warrants careful dose modifications to avoid adverse drug reactions or inadequate therapeutic responses [10,11]. However, inappropriate renal dose-adjustments, including drug doses that are not adjusted when indicated, incorrect adjustments, or contraindicated drugs, remain a common clinical problem in clinical practice [12]. Evidence suggests that healthcare providers often overlook kidney function when determining medication dosage, potentially resulting in adverse drug events, extended hospitalization, increased mortality, and further deterioration of renal function [12,13,14,15,16,17]. Although clear guidelines for renal dose-adjustment are available [18], compliance with these recommendations remains inadequate and is often attributed to multiple factors, including insufficient knowledge, limited clinical experience, or inadequate access to dosing resources. Furthermore, several patient-related factors, including advanced CKD stages (4, 5), polypharmacy, multiple comorbidities, and age > 65 years, are commonly linked to the prescription of inappropriate medication doses in individuals with CKD [11,19,20].

Patients with CKD face a markedly increased risk of cardiovascular complications. Approximately half of individuals with stage 4 to 5 CKD are affected by cardiovascular disease (CVD) [21], and cardiovascular-related mortality accounts for nearly 40–50% of deaths in patients with advanced CKD or end-stage kidney disease (ESRD), compared with approximately 26% among individuals with normal renal function [22,23]. Cardiovascular mortality in CKD is frequently attributable to heart failure and lethal arrhythmias, particularly in the later stages of the disease [24].

End-stage renal disease is becoming more prevalent in Saudi Arabia, with serious health implications [25]. According to the Saudi Center for Organ Transplantation (SCOT), 28,256 patients had ESRD in Saudi Arabia in 2019 [26]. Current estimates indicate that more than 20,000 individuals in the nation rely on hemodialysis to survive [12]. Furthermore, CVDs are expected to represent an even greater burden than currently recognized, particularly considering the ongoing increase in major risk factors, including obesity, diabetes, hypercholesterolemia, and hypertension, across the country [27].

Despite the high prevalence of CKD and its increased susceptibility to inappropriate medication dosing, particularly among those with coexisting CVDs, evidence regarding the prevalence of such dosing errors, contributing factors, and drugs most frequently involved in this high-risk population remains limited in Saudi Arabia. Previous studies were generally conducted with relatively small sample sizes and often focused on specific age groups, which restricts the generalizability of their findings across broader patient populations [12,28]. Consequently, the purpose of this study was to assess the prevalence of inappropriate medication dosing and explore factors associated with renal dose-adjustment outcomes among hospitalized patients with CKD and concomitant CVD in the Al-Baha region, Saudi Arabia. These findings will help identify targets for intervention (e.g., specific drugs or patient groups) and will contribute to the existing body of information, particularly in tertiary care settings.

## 2. Materials and Methods

This study was a retrospective, observational, cross-sectional analysis conducted via a systematic electronic medical record review. The study included patients admitted between January 2023 and December 2025 to two tertiary care hospitals within the Al-Baha region, Saudi Arabia: (a) King Fahad Referral Hospital (KFH), the primary, central hospital in the Al-Baha region under the Saudi Ministry of Health with a total bed capacity of 350, and (b) Prince Meshari bin Saud Hospital in Baljurashi (PMH), the second main hospital in the Al-Baha region under the Saudi Ministry of Health, with a 330-bed capacity.

Inclusion criteria were as follows: (a) Patients aged 18 years and above. (b) Confirmed diagnosis of both CVD (e.g., heart failure, ischemic heart disease, hypertension, dilated cardiomyopathy, cardiac valvular disease) and CKD (with eGFR < 60 mL/min, including dialysis patients). Patients were identified using the International Statistical Classification of Diseases and Related Health Problems, 10th Revision (ICD-10) (CKD: N18.1–N18.5, N18.9; CVD: I10–I16, I20–I25, etc.) [29]. To confirm chronicity, two estimated eGFR measurements < 60 mL/min/1.73 m^2^ documented at least three months apart were identified in the patients’ records. (c) Patients who were hospitalized for at least 24 h for any reason. (d) Patients receiving at least one medication with the potential to require renal dose-adjustment according to standard renal dosing references. The need for renal dose-adjustment was evaluated individually based on the patient’s CrCl at the time of prescribing. Patients were excluded if they (a) were admitted for less than 24 h or had incomplete medical information; (b) had acute kidney injury (AKI) or unstable kidney function; (c) had a single kidney or renal transplant; (d) did not have CVD or CKD; (e) were pregnant; or (f) died during hospitalization.

The required sample size was determined assuming a 95% confidence level, a 5% margin of error, and an expected prevalence of 48.3% based on prior literature [30]. Using these inputs, the minimum required sample size was 384 participants, estimated with the Raosoft^®^ online sample size calculator (Raosoft, Inc., Seatle, WA, USA).

The total screened records were 1479 (936 KFH + 543 PMH); 440 records were included, while 1039 records were excluded as follows: (a) 290: incomplete medical information, (b) 183: diagnosis of CKD only, (c) 98: AKI in addition to CKD, (d) 445: eGFR > 60 mL/min, (e) 11: neither CKD nor CVD, (f) 7: single kidney, and (g) 5: kidney transplant.

Relevant clinical and medication data were extracted from hospital records using a structured data abstraction form. The following information was recorded:

- Demographics: Age, sex, weight, and height for body mass index (BMI) calculation.
- Clinical data: Admission date, length of hospital stay, diagnosis, serum creatinine level, dialysis status, and comorbidities.
- Pharmacological: Prescribed medications, dose, and dosing schedule.

For each patient, CrCl was calculated based on the serum creatinine level recorded at the time of drug prescription using the Cockcroft-Gault formula [31] and used as the primary metric for renal dose-adjustment recommendations. eGFR values calculated using the CKD-EPI equation and indexed to 1.73 m^2^ were obtained from the patients’ medical records and used to classify patients into the five stages of CKD: stage 3a (45–59 mL/min), stage 3b (30–44 mL/min), stage 4 (15–29 mL/min), stage 5 (<15 mL/min).

Patients receiving dialysis were categorized separately as individuals undergoing renal replacement therapy rather than as an eGFR-defined CKD stage. All dialysis patients included in this study were receiving intermittent hemodialysis (HD); patients undergoing peritoneal dialysis were not identified in the cohort.

Medication lists were reviewed to identify drugs with potential for renal dosing considerations. The requirement for dose-adjustment was determined according to the patient’s CrCl using recommendations from the hospital’s drug formulary and Lexicomp^®^ Drug Information Handbook as confirmatory references [32]. Each medication’s recommended dose-adjustment was compared with the actual patient-prescribed dose, and its appropriateness was determined accordingly. For patients undergoing hemodialysis, dosing appropriateness was assessed using hemodialysis-specific dosing recommendations, considering drug dialytic clearance and recommended timing of administration relative to dialysis sessions, as specified in the hospital’s drug formulary and Lexicomp^®^ Drug Information Handbook. All data were entered into an Excel spreadsheet for further analysis. Prescribed drugs were categorized as: (a) appropriately adjusted, (b) inappropriately adjusted, (c) not adjusted, (d) no need for adjustment, and (e) contraindicated.

Descriptive statistics were used to summarize patient demographics, clinical characteristics, renal disease severity, and prescription patterns. Categorical variables were presented as frequencies and percentages. The primary unit of analysis for renal dose-adjustment outcomes was the prescription. Analyses were conducted at two levels. Patient demographic and clinical characteristics were summarized at the patient level (N = 440). Because individual patients could receive more than one eligible medication, renal dose-adjustment outcomes were evaluated at the prescription level (N = 787), with each eligible prescription assessed separately according to CrCl-based dosing recommendations. Renal dose-adjustment outcomes were classified into five categories: appropriate adjustment, inappropriate adjustment, not adjusted despite indication, no need for adjustment, and contraindicated use. These categories were mutually exclusive at the prescription level, as each prescription was assigned to only one category. Associations between patient-level characteristics and renal dose-adjustment outcomes derived from prescription-level classifications were assessed using Pearson’s chi-square test. Fisher’s exact test was used when the expected cell count was less than 5. Because these analyses did not adjust for within-patient clustering or adjust for potential confounders, the findings should be interpreted as exploratory associations rather than independent predictors. To account for multiple comparisons across renal dose-adjustment outcomes, *p*-values were adjusted using the Holm–Bonferroni method. All tests were two-sided, and Holm-adjusted *p*-values < 0.05 were considered statistically significant. Analyses were performed using IBM SPSS Statistics software (Version 21.0; IBM Corp., Armonk, NY, USA).

## 3. Results

### 3.1. Study Cohort Characteristics

A total of 440 patients were included in this analysis. The study cohort was predominantly older, with more than half of patients aged >65 years, whereas smaller proportions were aged 41–65 years, 31–40 years, and 18–30 years. The sex distribution showed a slight predominance in male patients, although both sexes were well represented. Most patients were classified as overweight or obese, whereas fewer patients fell within the normal or underweight categories. Patients were included from two hospitals, with a slightly higher proportion originating from PMH than KFH. Cases were relatively evenly distributed across the study years of 2023, 2024, and 2025, with no marked clustering in a single year. Detailed demographic and baseline characteristics of the study population are summarized in Table 1.

### 3.2. Clinical Profile and Renal Disease Severity

The study population exhibited substantial clinical complexity and advanced renal impairment. The mean length of hospital stay was 4.5 ± 4.9 days, with a median stay of 3 days (IQR: 2–6). Most patients had a hospital stay of 1–2 days (48.4%), followed by 3–6 days (31.6%), whereas fewer patients remained hospitalized for 7–15 days (15.7%) or more than 15 days (4.3%). Renal function was markedly impaired in this cohort. For non-dialysis patients, the mean eGFR calculated using the CKD-EPI equation and indexed to body surface area (mL/min/1.73 m^2^) was 28.8 ± 11.3 mL/min/1.73 m^2^, with a median of 24.5 mL/min/1.73 m^2^ (IQR: 15–34 mL/min/1.73 m^2^). Dialysis-dependent patients were excluded from this calculation, as eGFR equations are not validated in patients receiving renal replacement therapy and do not reflect the clearance provided by dialysis. Most patients were classified as having stage 4 chronic kidney disease (38.2%) or stage 3b disease (30.9%), whereas 11.4% had stage 5 disease. Additionally, 17.5% of patients were receiving dialysis at the time of prescription. A considerable number of patients required a high level of care. Intensive care unit admission was documented in 39.1% of patients, including 28.9% with one admission, 7.3% with two admissions, and 3.0% with more than two admissions. Comorbid conditions were highly prevalent. Hypertension was present in 95.5% of patients, while diabetes mellitus affected 78.6%. Other cardiovascular conditions included heart failure (16.6%), cerebrovascular accident (6.4%), and less frequent diagnoses, such as ischemic heart disease and cardiomyopathy. Non-cardiovascular comorbidities included hypothyroidism (14.8%), dyslipidemia (6.4%), asthma (5.5%), and interstitial lung disease (6.1%), among others. Most patients were admitted to internal medicine services (75.9%), followed by the intensive care unit (13.6%) and surgical wards (8.6%), with a smaller proportion managed in the coronary care unit (1.8%). A comprehensive summary of clinical characteristics and renal disease severity is presented in Table 2.

### 3.3. Distribution and Patterns of Renal Dose-Adjustment Outcomes

Across the study cohort (N = 440), renal dose-adjustment practices demonstrated substantial variability. Table 3 presents renal dose-adjustment outcomes primarily at the prescription level, where categories are mutually exclusive because each prescription was assigned to only one outcome. The patient-level summary reflects whether a patient had at least one prescription in a given category and is therefore not mutually exclusive. Overall, appropriate dose-adjustment was the most frequently observed outcome, followed by prescriptions in which no dose-adjustment was required based on renal function. Nevertheless, a considerable proportion of prescriptions were classified as not adjusted, despite an indication for dose modification, whereas smaller proportions reflected inappropriate adjustment or contraindicated medication use. Distinct patterns were observed when stratified according to therapeutic class (Table 3). Among the antibiotics, appropriate dose-adjustment was most frequently documented for piperacillin/tazobactam and vancomycin, whereas ceftazidime IV and ciprofloxacin demonstrated higher frequencies of inappropriate adjustment. Both oral and intravenous amoxicillin/clavulanate formulations exhibited mixed patterns, with notable proportions of prescriptions either appropriately adjusted or not adjusted when indicated. For anticoagulant therapy, enoxaparin accounted for a substantial number of renal dosing decisions, with many prescriptions categorized as not requiring dose-adjustment and a clinically relevant proportion classified as not adjusted at all or contraindicated (Table 3). Among the antidiabetic agents, metformin showed a heterogeneous distribution of outcomes, including appropriate adjustment, lack of adjustment when indicated, and contraindicated use. Sitagliptin prescriptions were predominantly categorized as not adjusted, whereas sulfonylureas demonstrated a higher frequency of appropriate adjustment in conjunction with notable contraindications (Table 3). Among cardiovascular agents, angiotensin-converting enzyme inhibitors were most associated with appropriate dose-adjustments, with relatively few instances of inappropriate or missed adjustments. However, spironolactone was frequently associated with contraindications. Among lipid-lowering agents, statins demonstrated a balanced distribution across appropriate adjustments, the absence of adjustment when indicated, and cases where no adjustment was required (Table 3). Polypharmacy, defined as the concurrent use of five or more medications, was present in 401 of 440 patients (91.1%). Among these patients, the number of evaluated medications per patient had a mean of 7.17 ± 1.89, a median of 7 (IQR: 6–8), and a range of 5–8. For the prescription-level analyses shown in Table 4, patients with polypharmacy contributed 572 prescriptions, whereas patients without polypharmacy contributed 49 prescriptions.

**Figure 1:**
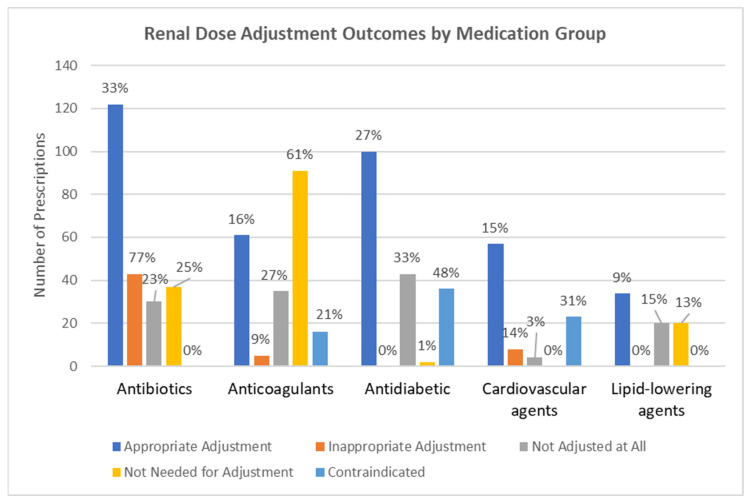
Comparison of renal dosing adjustment outcomes across medication groups at the prescription level. The chart displays renal dose-adjustment outcomes across the medication groups. The Y-axis indicates the number of prescriptions (*n*), and bar height represents the frequency of prescriptions falling into each renal dose-adjustment outcome category within a given medication group. The percentage (%) shown above each bar represents the proportion within the corresponding renal dose-adjustment outcome, calculated using the total number of that outcome across all medication groups as the denominator. Accordingly, the percentages quantify the contribution of each medication group to the overall burden of each renal dosing outcome. Percentages may not sum exactly to 100% because of rounding.

Figure 1 illustrates the variation in renal dose-adjustment outcomes across medication groups. As shown, considerable differences exist in dose optimization practices among therapeutic classes.

### 3.4. Patient and Prescription-Level Factors Associated with Renal Dose-Adjustment Outcomes

Associations between patient characteristics, clinical factors, and renal dose-adjustment outcomes are summarized in Table 4. Because multiple comparisons were performed across renal dose-adjustment outcomes, *p*-values were adjusted using the Holm–Bonferroni method. After adjustment, age was not significantly associated with any renal dose-adjustment outcome (Holm-adjusted *p* = 0.145), although descriptively higher proportions of unadjusted prescriptions were observed among older patients. Similarly, sex was not significantly associated with renal dose-adjustment outcomes after correction for multiple testing (Holm-adjusted *p* = 0.065). Although female patients accounted for a higher proportion of contraindicated medication use than male patients (58.5% vs. 41.5%), this difference did not remain statistically significant after adjustment.

Body mass index (BMI) demonstrated borderline statistical significance after correction (Holm-adjusted *p* = 0.05). Overweight and obese patients accounted for a larger proportion of prescriptions categorized as requiring no renal dose modification, while contraindicated prescriptions were also more frequently observed among obese patients. However, BMI was not significantly associated with appropriate or inappropriate dose- adjustment.

A significant association between hospital site and renal dose-adjustment outcomes remained after Holm correction (Holm-adjusted *p* = 0.01) in these exploratory bivariate analyses. Prescriptions from Prince Meshari Bin Saud Hospital (PMH) showed higher proportions of unadjusted prescriptions compared with King Fahad Hospital (KFH), indicating variability in prescribing practices between institutions.

The year of prescription was not significantly associated with renal dose-adjustment outcomes, suggesting relative stability in prescribing patterns over the study period. The frequency of intensive care unit (ICU) admissions was significantly associated with renal dose-adjustment outcomes after correction (Holm-adjusted *p* = 0.04). Patients without ICU admission accounted for the largest proportion of appropriately adjusted prescriptions, whereas increasing ICU admission frequency was associated with lower proportions of appropriate adjustments. ICU admission frequency was not significantly associated with inappropriate or contraindicated prescriptions.

Finally, chronic kidney disease (CKD) stage demonstrated a strong and statistically significant association with renal dose-adjustment outcomes (Holm-adjusted *p* < 0.005). Advanced CKD stages and dialysis status were associated with higher proportions of both appropriate dose-adjustments and contraindicated prescriptions, whereas earlier CKD stages accounted for a larger proportion of prescriptions categorized as not requiring adjustment. Polypharmacy was highly prevalent in the cohort but was not significantly associated with renal dose-adjustment outcomes after adjustment for multiple testing.

## 4. Discussion

In patients with renal impairment, all pharmacokinetic parameters, including drug bioavailability, protein binding, biotransformation, volume of distribution, and renal excretion, were altered. Hence, this requires carefully guided medication dosage adjustment to avoid drug toxicity or reduced therapeutic effectiveness, tailored to the estimated renal function, existing comorbid diseases, and concurrently prescribed medications [33].

Our study showed that at the prescription level, 48% of medications were appropriately adjusted, 7% were inappropriately adjusted, 17% were not adjusted despite an indication, 19% required no dose-adjustment, and 10% were contraindicated. At the patient level, 85% of patients had at least one appropriately adjusted medication, 13% had at least one inappropriately adjusted medication, 30% had at least one unadjusted medication, and 17% had at least one contraindicated prescription.

Notably, in exploratory bivariate analyses, patients with advanced CKD and those undergoing dialysis had a significantly higher rate of appropriate dose-adjustment prescriptions and contraindicated prescriptions: specifically, patients with stage 4 CKD had the highest rate of appropriate adjustments (60% of prescriptions), while dialysis patients had the highest rate of contraindicated prescribing (38.9% of prescriptions). This dual finding illustrates how difficult it is to manage patients with CKD and emphasizes the value of working with an interprofessional team that includes pharmacists in order to avoid, detect, and address medication-related issues [34,35]. In comparison, previous studies have reported higher rates of inappropriate dosing: Chertow et al. (2001) reported an inappropriate dosing rate of 70% [36], while Falconnier et al. (2001) reported that 67% of prescribed drugs were administered at inappropriate doses [37]. Similarly, high rates of unadjusted dosing were observed in Pakistan (58.2% and 59.58%), Lebanon (49%), and South Africa (68%) [19,38,39,40]. The relatively lower rate of inappropriate prescribing observed in our study can be explained by multiple factors. First, most patients in our study were in the latest stages of renal dysfunction (stages 3b, 4, and 5), with 17.5% undergoing dialysis. While these patients may receive more frequent nephrology consultations and strict adherence to medication prescription guidelines, clinicians may also encounter situations where the benefits of medications are weighed against contraindications, resulting in continued use despite renal impairment. Additionally, knowledge gaps regarding absolute contraindications in dialysis patients may contribute to this finding [41]. However, as we did not measure consultation frequency or guideline adherence directly, these explanations remain hypotheses requiring further investigation. Second, all hospitalized patients in our study presented with multiple comorbidities (95.5% with hypertension, 16.6% with heart failure, 78.6% with diabetes, etc.). Prescription decisions in such complex patients are challenging for physicians. We hypothesize that physicians may adopt a cautious approach by initiating treatment at the lowest therapeutic dose, with plans for up-titration during outpatient follow-up [30]. Our results are consistent with several studies reporting relatively low rates of inappropriate drug dosing (11.9%, 28.2%, and 35%) in the USA, Korea, and France, respectively [42,43,44].

In our study, antibiotics were the most common drug class that was inappropriately adjusted (77%), with IV ceftazidime and oral ciprofloxacin showing the most frequent inappropriately adjusted medications (80% and 60.9%, respectively). These findings are in line with previously published data. A study in Jordan found that 36.25% of CKD patients were prescribed antibiotics without dose-adjustments for renal function [45]. Similarly, antibiotic dosing errors were documented in 38.4% of hospitalized patients with renal impairment [46]. Aghaei et al. (2015) also reported a high proportion of unadjusted antibiotic doses (67%) [47]. Unlike other studies [48,49,50,51,52] that reported high rates of inappropriate dosing, our cohort demonstrated the highest rates of appropriate dose-adjustments for piperacillin/tazobactam and vancomycin. In those previous reports, inappropriate piperacillin/tazobactam dosing often resulted from the use of fixed regimens (e.g., 3.375 g every 6 h or 4.5 g every 6–8 h) rather than regimens (e.g., 2.25 g every 6–8 h) adjusted for CrCl and infection type, a practice that was implemented consistently in our study. Piperacillin/tazobactam is primarily excreted unchanged by the kidneys. Higher doses have been linked to reduced effectiveness and an increased risk of acute kidney injury. Therefore, lowering the dose will help preserve kidney function while effectively treating infection [53].

Among medication groups that were inappropriately prescribed in our study, ranging from not adjusted at all (33%) to contraindicated (48%), antidiabetics showed the highest proportion. Sitagliptin prescriptions showed the highest unacceptable malprescription frequency (100%, not adjusted at all), whereas 9.2% and 18.4% of metformin cases were not adjusted at all or contraindicated, respectively. In accordance with our findings, among other drug classes, antidiabetics were the most incorrectly administered [54]. According to additional research, all patients with an eGFR of less than 30 mL/min received an improper prescription for metformin [19,55]. Moreover, in Australia, sitagliptin was found to be prescribed at doses higher than needed in 22.55% of cases [56]. Typically, these medications are prescribed for patients with difficult-to-control diabetes. Therefore, after weighing the benefit/risk ratio, doctors tend to maintain patients at their current doses while monitoring for possible side effects. Physicians may also underestimate adverse effects, especially in patients with advanced renal impairment [19].

The underlying pathophysiological mechanisms of hypertension, heart disease, and CKD are closely related, which may explain the regular inappropriate prescription patterns of cardiovascular agents identified in both hospital and outpatient settings [57]. Relatively few occurrences of improper (13.8%) or missed dose modifications (5.2%) with perindopril were found in our study. Conversely, spironolactone was strongly linked to prohibited use (67.6%), whereas statins had a 27% missed adjustment rate. In line with our findings, Jamaluddin et al. (2023) found that although spironolactone was prescribed to fewer than 20 patients, most of them received the wrong dosage [58]. Similar results were reported in Ethiopia and Pakistan, where more than 50% of spironolactone prescriptions were administered without dose-adjustments [20,38]. In contrast, spironolactone had a low incorrect dosage rate (4.2%), with the majority of patients administered 25 mg daily; the highest permitted daily dose is 100 mg [30].

In the present study, enoxaparin prescriptions demonstrated a clinically higher percentage categorized as either contraindicated (7.7%) or not altered at all (16.8%), with the largest frequency falling into the proper dosage category (29.3%). The frequency of improper enoxaparin dosage in individuals with CKD has not been studied extensively. Nearly half of the 10,687 participants in a large-scale trial received improper doses, with a consequent increased risk of serious bleeding events and death [59]. Furthermore, the accuracy of enoxaparin-administered doses has been studied across various hospital types as well as for certain patient populations (severe renal insufficiency and extremely obese patients). Forty percent of the 463 participants received an incorrect dose of enoxaparin [60]. In addition to renal function status, which is considered an important factor in determining the correct enoxaparin dose, several other parameters are also critical, including age, body weight, and indication.

In our cohort, age was not significantly associated with any renal dose-adjustment outcome after Holm–Bonferroni correction (*p* = 0.145). While older patients (>65 years) descriptively accounted for the highest number of unadjusted prescriptions (*n* = 81), this did not reach statistical significance, suggesting that age alone was not a primary driver of dosing errors when compared to clinical factors like CKD stage. Although the statistical analysis showed no significant correlation, the observed frequencies could not be completely ignored. Multiple comorbidities, as well as incorrect eGFR estimation, which are commonly associated with this subset of patients, can lead to incorrect medications [20,61]. Other studies reported that 6–28% of geriatric patients and nursing home residents experience contraindicated drug use or require renal dosage adjustments [62]. Likewise, in Stockholm, Sweden, where a large population-based study was conducted, 43.6% of the screened sample (*n* = 30,372) aged 65 years or older and with a confirmed diagnosis of CKD stages 3 and 4 received inappropriately high doses of prescribed medications [63]. Regarding sex differences, and consistent with other studies [48,64], we did not find a significant correlation between sex and any of the renal dose-adjustment outcomes.

Obesity can alter the volume of distribution and clearance for many medications, and when combined with reduced renal function, the risk of prescribing medications that are inappropriate may increase [65]. In our cohort, BMI demonstrated borderline statistical significance (*p* = 0.05). Obese and overweight patients were significantly more likely to receive prescriptions categorized as requiring no adjustment, and obese patients had the highest proportion of contraindicated medications. However, its lack of association with appropriate or inappropriate adjustment suggests its role is secondary to disease severity.

Our results revealed that the appropriate prescriptions for patients with no ICU admission were significantly higher than those for patients whose conditions required ICU admission. The proportion of appropriately adjusted prescriptions decreased as ICU admission frequency increased from 50% among patients with no ICU admissions to 41.2% among those with more than two admissions. Importantly, we observed no significant association between either inappropriate or contraindicated prescribing and repeated ICU admissions, although the proportion of appropriately adjusted prescriptions declined with increasing ICU admissions. This likely reflects the greater clinical complexity of patients requiring repeated intensive care rather than a decline in the prescribing quality itself. Whether this pattern affected patient safety cannot be determined from our data, as we did not measure clinical outcomes such as adverse drug events. Consistently, it was reported that patients admitted to the CCU had higher rates of inadequate adjustment [39]. A systematic review reported that nonadherence to renal dosing guidelines ranges from 19% to 69% in all contexts, with ambulatory care showing the highest prevalence [66]. Another review also noted that outpatient settings had a higher prevalence of renally inappropriate drug usage (1–37%) [67]. The significant variation in prescription practices between the two hospitals was one of our study’s key findings. Prescriptions from PMH showed higher proportions of unadjusted medications (73/341 prescriptions, 21.4%) compared to KFH (43/280 prescriptions, 15.4%), while KFH had more prescriptions requiring no adjustment (64/280, 22.9% vs. 46/341, 13.5% at PMH). Our results are consistent with earlier research. In Bangladesh, associations between hospital type and prescribing errors were reported, with polypharmacy rates varying from 49.5% to 71.0% across institutions [68]. Additionally, the rate of incorrect prescribing ranged from 12.6% to 96% in various hospital settings, according to a recent systematic study, providing more evidence that institutional characteristics significantly affect the quality of prescriptions [69]. Our observation might be explained by variations in clinical pharmacy services, decision support tools, differences in prescribers’ awareness, or unmeasured patient characteristics. This finding highlighted the necessity of focused quality improvement at the institutional level. Unfortunately, the purpose of our study was not to pinpoint the precise causes of this variability.

Polypharmacy was not significantly associated with any dosing outcome in our bivariate exploratory analyses. This finding may be partially explained by the extremely high prevalence of polypharmacy in our cohort (*n* = 572), compared with a small number of prescriptions contributed by patients without polypharmacy (*n* = 49), which limits the statistical power to detect differences between groups. While previous studies have reported multiple medications as a strong factor associated with inappropriate renal prescribing [70,71,72], the present results should be interpreted as exploratory bivariate observations that were not adjusted for confounding or within-patient clustering.

Strengths: To our knowledge, this study was the first to be performed in the Al-Baha region and one of the few undertaken in Saudi Arabia addressing this issue. Additionally, instead of investigating prescription errors reported by practitioners, this study evaluated all included medications by reviewing medical records on an individual basis. This type of detection yields a more accurate assessment of error magnitude compared to opportunistic incident reporting, which often underestimates error prevalence. On the other hand, several limitations should be acknowledged. The retrospective cross-sectional design is inherently vulnerable to incomplete documentation and missing data, which may have influenced the classification of prescribing appropriateness. Nevertheless, the study reflects routine clinical practice and enabled the inclusion of all eligible patients identified during the study period. Future multicenter studies with longitudinal follow-up are warranted to better delineate determinants of inappropriate prescribing and to strengthen causal inference.

## 5. Conclusions

Overall, in our study, most prescriptions were consistent with recommended renal dosing; however, clinically relevant instances of suboptimal prescribing were observed across the included clinical settings.

Our findings have several important clinical implications. Several regularly prescribed drugs, including metformin, sitagliptin, ceftazidime, ciprofloxacin, and spironolactone, were prescribed inappropriately to patients with CKD. In exploratory bivariate analyses, age showed descriptive trends toward higher unadjusted prescriptions but lacked statistical significance after Holm–Bonferroni correction for multiple comparisons (adjusted *p* = 0.145). BMI showed borderline correlation with renal dose-adjustment outcomes (adjusted *p* = 0.05), though this finding should be interpreted cautiously given the exploratory nature of the analyses and the lack of adjustment for potential confounders. Polypharmacy, though highly prevalent in our cohort, was not significantly associated with any dosing outcome in these bivariate analyses, likely due to limited statistical power resulting from its near-universal presence in the cohort. These findings represent exploratory bivariate associations only and require confirmation in future studies employing multivariable regression models that adjust for within-patient clustering and adjust for potential confounding variables.

Clinicians should be aware of the appropriate dosages of these medications and consider these factors when prescribing them to patients with CKD. These findings underscore the importance of routinely incorporating kidney function assessment into prescribing decisions and reinforcing adherence to renal dosing guidance for frequently used medications in patients with CKD. Furthermore, pharmacists must play a key role in optimizing prescriptions and safely adjusting medication doses for patients with CKD.

## Figures and Tables

**Table 1:** Sociodemographic profile of the study cohort.

	Mean ± SD	Median (IQR)
**Age** (years)	67.89 ± 15.12	69 (58–79)
	**Frequency**	**Percentage**
**Age** (years)		
18–30	6	1.4%
31–40	15	3.4%
41–65	158	35.9%
>65	261	59.3%
**Sex**		
Female	195	44.3%
Male	245	55.7%
**BMI**		
Underweight	9	2%
Normal	114	25.9%
Overweight	173	39.3%
Obese	144	32.7%
**Hospital**		
KFH	201	45.7%
PMH	239	54.3%
**Year**		
2023	132	30%
2024	164	37.3%
2025	144	32.7%

**Table 2:** Clinical profile, disease severity, and comorbidity burden.

		Mean ± SD		Median (IQR)			
**Length of Hospital Stay** (Days)		4.5 ± 4.9		3 (2–6)			
* eGFR (mL/min/1.73 m^2^)		28.8 ± 11.3		24.5 (15–34)			
		**n**	**%**			***N***	**%**
**Length of Hospital Stay** (Days)				**Intensive Care Unit Admissions**			
	1 to 2	213	48.4%		0 admissions	268	60.9%
	3 to 6	139	31.6%		1 admission	127	28.9%
	7 to 15	69	15.7%		2 admissions	32	7.3%
	>15	19	4.3%		>2 admissions	13	3%
**CKD stage (eGFR** [mL/min/1.73 m^2^])				**Comorbidities**			
	Stage 3a (45–59)	9	2%		Diabetes Mellitus (DM)	346	78.6%
	Stage 3b (30–44)	136	30.9%		Hypothyroidism	65	14.8%
	Stage 4 (15–29)	168	38.2%		Dyslipidaemia	28	6.4%
	Stage 5 (<15)	50	11.4%		Interstitial Lung Disease	27	6.1%
	**Dialysis (Separate Category)**	77	17.5%		Asthma	24	5.5%
**Cardiovascular Diseases (CVDs)**					Hyperuricemia	14	3.2%
	Hypertension	420	95.5%		Chronic Obstructive Pulmonary Disease (COPD)	13	3%
	Heart Failure (HF)	73	16.6%		Liver Cirrhosis	10	2.3%
	Ischemic Heart Disease (IHD)	1	0.2%		Hyperthyroidism	8	1.8%
	Dilated Cardiomyopathy	8	1.8%		Malignancy (active or treated)	8	1.8%
	Cardiac Valvular Disease	8	1.8%		Epilepsy	7	1.6%
	Pulmonary Hypertension	8	1.8%		Smoker	7	1.6%
	Cerebrovascular Accident	28	6.4%		Morbid Obesity	6	1.4%
	Arrythmia	0	0%		Hepatitis B Virus (HBV)	4	0.9%
	Hypertensive Heart Disease (HHD)	0	0%		Heavy Smoker	2	0.5%
	Peripheral Venous Disease (PVD) or DVT	0	0%		Hepatitis C Virus (HCV)	2	0.5%
	Peripheral Arterial Disease (PAD)	2	0.5%		Preeclampsia	2	0.5%
**Department**					Sickle Cell Anemia	2	0.5%
	Coronary Care Unit	8	1.8%		Others	16	3.6%
	Intensive Care Unit	60	13.6%				
	Internal Medicine	334	75.9%				
	Surgical Ward	38	8.6%				

* mean eGFR values are reported for non-dialysis patients only (*n* = 363). Dialysis patients (*n* = 77) are excluded from this calculation, as eGFR is not validated in this population.

**Table 3:** Distribution of renal dose-adjustment outcomes across medication classes and individual drugs, showing both patient-level summaries (N = 440) and prescription-level summaries (N = 787).

Medication Group	Drug	*n*	%	Renal Dose-Adjustment Categories				Contraindicated
				Appropriate Adjustment	Inappropriate Adjustment	Not Adjusted at All	Not Needed for Adjustment	
**Overall** (patients, N = 440)		440		374 (85%)	56 (13%)	132 (30%)	150 (34%)	75 (17%)
**Overall** (prescriptions, N = 787)		787		374 (48%)	56 (7%)	132 (17%)	150 (19%)	75 (10%)
**Antibiotics**	Amoxicillin/clavulanate	64	14.5	26 (40.6%)	0 (0.0%)	15 (23.4%)	23 (35.9%)	0 (0.0%)
	Amoxicillin/clavulanate IV	11	2.5	6 (54.5%)	0 (0.0%)	3 (27.3%)	2 (18.2%)	0 (0.0%)
	Cefuroxime	32	7.3	8 (25.0%)	1 (3.1%)	11 (34.4%)	12 (37.5%)	0 (0.0%)
	Ceftazidime IV	35	8.0	6 (17.1%)	28 (80.0%)	1 (2.9%)	0 (0.0%)	0 (0.0%)
	Piperacillin/tazobactam	49	11.1	49 (100.0%)	0 (0.0%)	0 (0.0%)	0 (0.0%)	0 (0.0%)
	Ciprofloxacin	23	5.2	9 (39.1%)	14 (60.9%)	0 (0.0%)	0 (0.0%)	0 (0.0%)
	Vancomycin	18	4.1	18 (100.0%)	0 (0.0%)	0 (0.0%)	0 (0.0%)	0 (0.0%)
	**Overall**	232		122 (53%)	43 (19%)	30 (13%)	37 (16%)	0 (0%)
**Anticoagulants**	Enoxaparin	208	47.3	61 (29.3%)	5 (2.4%)	35(16.8%)	91 (43.8%)	16 (7.7%)
**Antidiabetic**	Metformin	98	22.3	69 (70.4%)	0 (0.0%)	9 (9.2%)	2 (2.0%)	18 (18.4%)
	Sitagliptin	34	7.7	0 (0.0%)	0 (0.0%)	34 (100.0%)	0 (0.0%)	0 (0.0%)
	Gliclazide	49	11.1	31 (63.3%)	0 (0.0%)	0 (0.0%)	0 (0.0%)	18 (36.7%)
	**Overall**	181		100 (55%)	0 (0%)	43 (24%)	2 (1%)	36 (20%)
**Cardiovascular agents**	Angiotensin-converting enzyme inhibitors (ACEI) (perindopril)	58	13.2	47 (81.0%)	8 (13.8%)	3 (5.2%)	0 (0.0%)	0 (0.0%)
	Spironolactone	34	7.7	10 (29.4%)	0 (0.0%)	1 (2.9%)	0 (0.0%)	23 (67.6%)
	**Overall**	92		57 (62%)	8 (9%)	4 (4%)	0 (0%)	23 (25%)
**Lipid-lowering agents**	Statins (rosuvastatin)	74	16.8	34 (45.9%)	0 (0.0%)	20(27.0%)	20 (27.0%)	0 (0.0%)
	**Polypharmacy**							
	Yes	401	91%					
	No	39	8.9%					

Values are presented as *n* (%). Percentages for individual drugs were calculated using the drug-specific denominator (*n*). The “Overall (per prescription)” row summarizes renal dose-adjustment outcomes across all prescriptions (N = 787), with each prescription assigned to one mutually exclusive category. The “Overall (per patient)” row summarizes the proportion of patients (N = 440) who had at least one prescription in a given category; therefore, these categories are not mutually exclusive, and the cumulative percentage may exceed 100%. Category definitions were as follows: appropriate adjustment (correct dose modification based on renal function); inappropriate adjustment (incorrect dose modification); not adjusted at all (no modification despite indication); no adjustment required (correct dosing without modification); and contraindicated (use despite renal contraindication). Percentages may not sum exactly to 100% because of rounding.

**Table 4:** Distribution of renal dose-adjustment outcomes at the prescription level across patient demographic and clinical characteristics.

	Total Prescriptions in Category (*n*)	Appropriate Adjustment	*p*-Value	Inappropriate Adjustment	*p*-Value	Not Adjusted at All	*p*-Value	Not Needed for Adjustment	*p*-Value	Contraindicated	*p*-Value	Holm-Adjusted *p*-Value
**Age (years)**												
18–30	9	6 (66.7%)	0.272	0 (0.0%)	0.296	0 (0.0%)	0.029	1 (11.1%)	0.067	2 (22.2%)	0.343	0.145
31–40	20	10 (50.0%)		2 (10.0%)		2 (10.0%)		3 (15.0%)		3 (15.0%)		
41–65	213	103 (48.4%)		7 (3.3%)		33 (15.5%)		51 (23.9%)		19 (8.9%)		
>65	379	183 (48.3%)		19 (5.0%)		81 (21.4%)		55 (14.5%)		41 (10.8%)		
**Sex**												
Female	289	130 (45.0%)	0.427	12 (4.2%)	0.872	55 (19.0%)	0.434	54 (18.7%)	0.245	38 (13.1%)	0.013	
Male	332	172 (51.8%)		16 (4.8%)		61 (18.4%)		56 (16.9%)		27 (8.1%)		0.065
**BMI**												
Underweight	17	7 (41.2%)	0.273	2 (11.8%)	0.123	4 (23.5%)	0.34	0 (0.0%)	0.01	4 (23.5%)	0.041	0.05
Normal	155	76 (49.0%)		9 (5.8%)		26 (16.8%)		25 (16.1%)		19 (12.3%)		
Overweight	213	107 (50.2%)		6 (2.8%)		35 (16.4%)		49 (23.0%)		16 (7.5%)		
Obese	236	112 (47.5%)		11 (4.7%)		51 (21.6%)		36 (15.3%)		26 (11.0%)		
**Hospital**												
KFH	280	139 (49.6%)	0.83	8 (2.9%)	0.06	43 (15.4%)	0.03	64 (22.9%)	0.002	26 (9.3%)	0.319	0.01
PMH	341	163 (47.8%)		20 (5.9%)		73 (21.4%)		46 (13.5%)		39 (11.4%)		
**Year**												
2023	189	88 (46.6%)	0.526	11 (5.8%)	0.341	39 (20.6%)	0.264	33 (17.5%)	0.87	18 (9.5%)	0.403	
2024	231	110 (47.6%)		7 (3.0%)		46 (19.9%)		39 (16.9%)		29 (12.6%)		
2025	201	104 (51.7%)		10 (5.0%)		31 (15.4%)		38 (18.9%)		18 (9.0%)		
**Intensive Care Unit Admissions**												
0 admissions	396	198 (50.0%)	0.01	13 (3.3%)	0.377	64 (16.2%)	0.307	89 (22.5%)	<0.001	32 (8.1%)	0.125	
1 admission	171	81 (47.4%)		12 (7.0%)		41 (24.0%)		15 (8.8%)		22 (12.9%)		0.04
2 admissions	37	16 (43.2%)		2 (5.4%)		7 (18.9%)		4 (10.8%)		8 (21.6%)		
>2 admissions	17	7 (41.2%)		1 (5.9%)		4 (23.5%)		2 (11.8%)		3 (17.6%)		
* **Polypharmacy**												
Yes	572	278 (48.6%)	0.317	26 (4.5%)	0.539	109 (19.1%)	0.212	100 (17.5%)	0.923	59 (10.3%)	0.91	
No	49	24 (49.0%)		2 (4.1%)		7 (14.3%)		10 (20.4%)		6 (12.2%)		
**CKD stage (eGFR (mL/min/1.73m^2^))**												
Stage 3a (45–59)	10	5 (50.0%)	<0.001	0 (0.0%)	0.872	2 (20.0%)	0.001	3 (30.0%)	<0.001	0 (0.0%)	<0.001	<0.005
Stage 3b (30–44)	241	100 (41.5%)		7 (2.9%)		28 (11.6%)		106 (44.0%)		0 (0.0%)		
Stage 4 (15–29)	225	135 (60.0%)		12 (5.3%)		54 (24.0%)		0 (0.0%)		24 (10.7%)		
Stage 5 (<15)	73	35 (47.9%)		4 (5.5%)		21 (28.8%)		0 (0.0%)		13 (17.8%)		
**Dialysis**	72	27 (37.5%)		5 (6.9%)		11 (15.3%)		1 (1.4%)		28 (38.9%)		

* Polypharmacy was defined as the concurrent use of five or more medications by a single patient. The 401 patients with polypharmacy contributed 572 prescriptions to the analysis; the 39 patients without polypharmacy contributed 49 prescriptions. Values are presented as n/N (%), where n is the number of prescriptions in a renal dose-adjustment category, and N is the total number of prescriptions within the corresponding subgroup. Percentages were calculated within each subgroup. Renal dose-adjustment outcomes were assigned at the prescription level, and each prescription was counted once only in one mutually exclusive category: appropriate adjustment, inappropriate adjustment, not adjusted at all, no adjustment required, or contraindicated. *p*-values were obtained using Pearson’s chi-square or Fisher’s exact test as appropriate. To account for multiple comparisons across renal dose-adjustment outcomes, *p*-values were adjusted using the Holm–Bonferroni method. Adjusted *p*-values ≥ 0.05 were considered not statistically significant.

## Data Availability

The raw data supporting the conclusions of this study will be made available by the authors upon request.

## Abbreviations

The following abbreviations are used in this manuscript:

CKD	Chronic kidney disease
CVD	Cardiovascular disease
ESRD	End-stage kidney disease
SCOT	Saudi Center for Organ Transplantation
KFH	King Fahad Referral Hospital
PMH	Prince Meshari Bin Saud Hospital
AKI	Acute kidney injury
BMI	Body mass index
CrCl	Creatinine clearance
ACEIs	Angiotensin-converting enzyme inhibitors
